# Wastewater-based reproduction numbers and projections of COVID-19 cases in three areas in Japan, November 2021 to December 2022

**DOI:** 10.2807/1560-7917.ES.2024.29.8.2300277

**Published:** 2024-02-22

**Authors:** Shogo Miyazawa, Ting Sam Wong, Genta Ito, Ryo Iwamoto, Kozo Watanabe, Michiel van Boven, Jacco Wallinga, Fuminari Miura

**Affiliations:** 1 Data Science Department, Shionogi and Co, Ltd, Osaka, Japan; 2 AdvanSentinel Inc., Osaka, Japan; 3 SHIMADZU Corporation, Kyoto, Japan; 4 Integrated Disease Care Division, Shionogi and Co, Ltd, Osaka, Japan; 5 Center for Marine Environmental Studies (CMES), Ehime University, Ehime, Japan; 6 Centre for Infectious Disease Control, National Institute for Public Health and the Environment (RIVM), Bilthoven, the Netherlands; 7 Julius Center for Health Sciences and Primary Care, University Medical Center Utrecht, Utrecht University, Utrecht, the Netherlands; 8 Department of Biomedical Data Sciences, Leiden University Medical Center, Leiden, the Netherlands

**Keywords:** Wastewater surveillance, wastewater based-epidemiology, scenario modelling, reproduction number, disease monitoring

## Abstract

**Background:**

Wastewater surveillance has expanded globally as a means to monitor spread of infectious diseases. An inherent challenge is substantial noise and bias in wastewater data because of the sampling and quantification process, limiting the applicability of wastewater surveillance as a monitoring tool.

**Aim:**

To present an analytical framework for capturing the growth trend of circulating infections from wastewater data and conducting scenario analyses to guide policy decisions.

**Methods:**

We developed a mathematical model for translating the observed SARS-CoV-2 viral load in wastewater into effective reproduction numbers. We used an extended Kalman filter to infer underlying transmissions by smoothing out observational noise. We also illustrated the impact of different countermeasures such as expanded vaccinations and non-pharmaceutical interventions on the projected number of cases using three study areas in Japan during 2021–22 as an example.

**Results:**

Observed notified cases were matched with the range of cases estimated by our approach with wastewater data only, across different study areas and virus quantification methods, especially when the disease prevalence was high. Estimated reproduction numbers derived from wastewater data were consistent with notification-based reproduction numbers. Our projections showed that a 10–20% increase in vaccination coverage or a 10% reduction in contact rate may suffice to initiate a declining trend in study areas.

**Conclusion:**

Our study demonstrates how wastewater data can be used to track reproduction numbers and perform scenario modelling to inform policy decisions. The proposed framework complements conventional clinical surveillance, especially when reliable and timely epidemiological data are not available.

**Key public health message**

**What did you want to address in this study and why?**

Wastewater surveillance is a promising tool for monitoring the spread of infectious diseases, but individual measurements of viral load in wastewater are inherently noisy. We proposed a modelling framework to capture the increasing or decreasing trend of a COVID-19 epidemic and provide scenario analyses to guide policy decisions.

**What have we learnt from this study?**

Epidemic trends were well-captured by the proposed modelling approach, using wastewater data alone. Estimated transmission parameters were comparable when using either reported case or wastewater data. This study also demonstrated how our approach can anticipate the potential impact of interventions on the expected case number over several months, using three study areas in Japan as a use case.

**What are the implications of your findings for public health?**

Wastewater surveillance complements existing surveillance systems. Given current changes in testing policies in many countries, reliable and timely epidemiological data would not always be available. As an alternative source of information, wastewater data, with appropriate data analysis, can support strategic planning and decision-making in public health.

## Introduction

The COVID-19 pandemic has presented a multifaceted challenge for policymakers to navigate, because of its complex dynamics influenced by vaccination, the emergence of new severe acute respiratory syndrome coronavirus 2 (SARS-CoV-2) virus variants and seasonality. Mathematical modelling has been employed by regional and national governments to monitor the disease in real-time, forecast epidemiological situations in the near future, e.g. 1–2 weeks ahead, and inform policy decisions by projecting long-term trajectories under different scenarios [1,2]. Scenario modelling, exemplified by various research groups such as the COVID-19 scenario hubs in the United States and Europe [3,4], has contributed to more realistic and robust projections and a better understanding of epidemiological characteristics of SARS-CoV-2. Accurate and standardised surveillance data are essential to capture temporal changes in disease dynamics and to provide input parameters for modelling analyses. However, it has become more challenging to obtain timely and unbiased epidemiological data via (passive) clinical surveillance because of changes in testing policies in many countries [5,6].

Wastewater surveillance has re-emerged as an alternative source of information during the COVID-19 pandemic [7,8]. Wastewater has the potential to monitor disease prevalence by measuring virus concentrations excreted by infected individuals, which does not rely on patients’ symptoms or medical-seeking behaviour [8,9]. The effectiveness of wastewater monitoring has been demonstrated for various infectious diseases (e.g. polio, mpox) in the past [8,10,11], and the COVID-19 pandemic has accelerated its establishment in many countries [7,12]. A remaining challenge inherent to wastewater surveillance is the substantial bias and noise in observed data because of the factors related to the sampling and quantification processes, e.g. higher water demand during daytime, dilution due to rainfall, PCR inhibition. To mitigate such biases, new molecular tools and sampling techniques have been developed [13]. Nevertheless, there remains the intrinsic noise in the observation process, and thus extracting true signals of epidemic growth requires data analytic methods that can disentangle underlying trends from noisy data.

Previous studies have attempted to deal with the noise in wastewater data by using statistical or machine-learning-based approaches [14-17]. The strength of these methods lies in the functional flexibility of models, which allows for the smoothing of noisy data, e.g. penalised splines [16], neural networks [14,17]. These studies primarily focused on short-term forecasting and aimed at providing near real-time estimates [15,16]. However, a drawback of non-mechanistic models is that they do not necessarily provide biological interpretations, and thus the outputs from such analyses are difficult to use for policy guidance with further scenario analysis.

Mechanistic models have been applied to wastewater data in recent studies, with the primary aim of evaluating the predictive ability of models [18,19] or monitoring growth trends by computing effective reproduction numbers [20,21]. Yet, another important component, scenario modelling, has not been thoroughly explored in combination with wastewater surveillance. Synthesis of multiple data streams would enhance the robustness of scenario modelling, and more importantly, there is a practical need to inform policymakers of strategic planning of interventions even in the absence of timely and reliable epidemiological data. In the current near-endemic situation of COVID-19, evaluating the potential impact of additional interventions such as vaccination campaigns is one of the key questions, even though notified data are not always fully available [5,6]. To this end, we need to exploit wastewater data and incorporate current transmission mechanisms, e.g. repeated infections related to emerging variants and waning immunity, which have not been explicitly captured in previous work [18,19].

In this study, we develop a modelling approach that accounts for reinfection and vaccination effects and propose a way to infer transmission parameters from wastewater data and integrate them with the scenario modelling framework. As a motivating example, we conducted wastewater monitoring in Japan and applied the proposed modelling approach to the collected wastewater and notified case data, in order to illustrate how wastewater data can be used for monitoring growth trends, short-term forecasting and scenario analysis.

## Methods

### Wastewater data

We implemented wastewater surveillance between November 2021 and December 2022, where there was sufficient access to confirmation testing during the SARS-CoV-2 Omicron wave. Wastewater monitoring was conducted in three study areas in Japan; Kyoto city (sewered population size: 778,000), a part of Kanagawa (sewered population size: 1,241,200, a subdistrict of Kanagawa city), and City A (sewered population size: 157,000). Wastewater samples were collected 2–3 times per week, and virus concentration in each sample was subsequently quantified with two different molecular methods, i.e. EPISENS-S and COPMAN [22,23]. The details of sampling methods and experimental procedures are provided in the Supplementary Text.

We normalised the observed SARS-CoV-2 concentration by a commonly used faecal indicator, i.e. Pepper mild mottle virus (PMMoV), to adjust for potential bias caused by sampling time and flow rate of influent wastewater. When the measured concentrations were below detection limits, we imputed them as 1 (copy/L) for computational convenience. We then constructed the time series of the normalised SARS-CoV-2 concentration by taking the geometric mean of individual raw RNA measurements on each day and the data was used for further analysis. All data sources and their availability are summarised in Supplementary Table S1. Observed wastewater and collected daily case data are illustrated in Supplementary Figure S2. Analysed data with permissions from municipalities are available in a GitHub repository (https://github.com/AdvanSentinel/AS-SEIRS).

### Epidemiological data

The number of daily confirmed cases within the same periods of wastewater sampling was obtained from the corresponding local government websites and a list of links to data sources is provided in Supplementary Table S1. As the coverage of the wastewater treatment plants does not always match the municipality areas, we calculated the daily number of cases in each catchment area by aggregating case data from multiple municipalities and weighting them by the proportion of the connected population size in each service area.

### Transmission model

We developed a compartmental SEIRS (susceptible-exposed-infectious-recovered and returning flow to susceptible due to immunity loss) model to incorporate reinfections and viral shedding from infected individuals to wastewater, adapting the method of Proverbio et al. [18]. The disease states susceptible S(t), exposed but not yet infectious E(t), infectious I(t) and recovered R(t) are depicted in the conceptual model diagram in Supplementary Figure S1. The model considered reinfections among individuals who have been infected already, by defining the average duration of immunity 1/ω that was assumed to be 180 days, i.e. recovered transition back to the susceptible state at the rate of ω[24]. We assumed fixed values for the mean latent period (1/α) of 1.5 days referring to the start of infectious viral shedding of the Omicron variant [25] and mean infectious period (1/τ) of 2 days to set the mean generation time as 3.5 days (based on the estimated mean serial interval [26]) in the main analysis. These parameters are summarised in Supplementary Table S2. The robustness of model fits to the change in these fixed parameters were checked with additional sensitivity analyses which are provided in Supplementary Figure S3. Three other parameters, the mean duration of virus shedding (1/γ), the scaling parameter for observed virus concentration (ν), and the time-varying transmission rate (β(t)), were estimated by fitting the model to daily cases and/or wastewater data; details can be found in Supplementary Materials. Here we employed a constant virus shedding rate γ, leading to the assumption that we can approximate the temporal variation in virus shedding by an exponential distribution.

### Stochastic SEIRS model and observation process

To estimate parameters, we implemented the above model as a stochastic model and calibrated it using an extended Kalman filter. The Kalman filter and extended filtering methods have been often used for calibrating a dynamic model with epidemiological surveillance data such as the daily number of reported cases [27,28]. In this study, we employed the filtering method to fit the transmission model to the observed daily cases, virus concentrations in wastewater, or both, by incorporating the observation errors. Details of calibration, model fit, and state-update steps are described in the Supplementary Materials.

The model dynamics, including the active virus shedding state A(t) and the (stochastic) observation errors, was described in the following system:


$$\frac{d}{dt}E\left(t\right)=\frac{\beta \left(t\right)S\left(t\right)I\left(t\right)}{N}-\alpha E\left(t\right)+\sqrt{\frac{\beta \left(t\right)S\left(t\right)I\left(t\right)}{N}}w_{1}\left(t\right)-\sqrt{\alpha E\left(t\right)}w_{3}\;(t)$$

$$\frac{d}{dt}I\left(t\right)=\alpha E\left(t\right)-\tau I\left(t\right)+\sqrt{\alpha E\left(t\right)}w_{3}\left(t\right)-\sqrt{\tau I\left(t\right)}w_{4}\;(t)$$

$$\frac{d}{dt}R\left(t\right)=\tau I\left(t\right)-\omega R\left(t\right)+\sqrt{\tau I\left(t\right)}w_{4}\left(t\right)-\sqrt{\omega R\left(t\right)}w_{2}\;(t)$$

$$\frac{d}{dt}A\left(t\right)=\frac{\beta \left(t\right)S\left(t\right)I\left(t\right)}{N}-\gamma A\left(t\right)+\sqrt{\frac{\beta \left(t\right)S\left(t\right)I\left(t\right)}{N}}w_{1}\left(t\right)-\sqrt{\gamma A\left(t\right)}w_{5}\;\left(t\right),$$

where the w_i_ are mutually independent white noise processes. We assume a closed population of size N and thus N = S(t)+E(t)+I(t)+R(t). The transitions here are assumed to follow a binomial process, and the binomial distribution is approximated by the normal distribution (see details in Proverbio et al. [18]). The model outputs were then compared with the observed data, i.e. case data or wastewater, or both.

For the observation process, we firstly assumed that the number of daily confirmed cases y_c_(t) is a fraction of infected individuals who newly become symptomatic on the date of observation


$$y_{C}\left(t\right)=\mu_{t}\int_{t-1}^{t}\alpha E\left(s\right)ds,$$

where μ_t_ is the reporting rate of newly confirmed cases of the total number of infected cases on the observation day t. Since μ_t_ may change depending on the day of the week and the national holidays, the day-of-week effect was adjusted, and the holiday effect was further incorporated by reducing the reporting rate by 75% based on the observed maximum change in testing rates in Tokyo during December 2022 [29]. Detailed computation process of μ_t_ is provided in the Supplementary Text. Secondly, the virus concentration in wastewater y_w_(t) is assumed to be proportional to the number of individuals shedding viruses A(t):


$$y_{w}\left(t\right)=\nu A\left(t\right).$$

In this equation, ν is a scaling parameter specific to study regions.

### Effective reproduction number

To quantify the growth trend of an epidemic, the (instantaneous) effective reproduction number [30,31], the number of secondary infections caused by a single infected person at time t, is calculated. In this study, the effective reproduction number is obtained by the following equation:


$$R_{eff}\left(t\right)=\frac{\beta \left(t\right)}{\tau }\frac{S\left(t\right)}{N}\;,$$

where the transmission rate β(t) is obtained by fitting the model to either notified case data or wastewater data. To distinguish between two different reproduction numbers, hereafter we use notification-based reproduction numbers


$$R_{eff}^{N}(t)$$

and wastewater-based reproduction numbers 


$$R_{eff}^{W}(t)$$

for further comparison. We computed the uncertainty in reproduction numbers by using estimates of β(t) and its standard deviation (SD) and visualised the uncertainty ranges of 2 SDs.

As a reference to standard practice, we used the EpiEstim package [32] to estimate effective reproduction numbers from notified case data, assuming a serial interval is gamma-distributed with a mean of 3.5 days and a SD of 2.4 days, i.e. the same mean generation as the main analysis with Kalman filter [26]. The EpiEstim estimators were then compared with the values computed by our approach.

### Forecasting and scenario projections

The model fitting via the Kalman filter allows an adaptive estimation of transmission rate β(t) at each time point, and thus we sequentially updated the estimated parameters using the most recent data points. To perform 1-week ahead forecasting, we simulated daily reported cases over the next 7 days using the most recent estimates of transmission rates and the number of individuals remaining in each state. The 1-week ahead prediction accuracy was evaluated by two error metrics (the root-mean-square error (RMSE) and the mean-absolute-error (MAE)).

We examined two intervention scenarios; increasing vaccination coverage and reducing contact rates by non-pharmaceutical interventions (NPIs). Initial conditions for projections were determined using the estimated number of individuals in each state by fitting the model to the most recent observed data. The study periods of observed data are described in Supplementary Table S1, and the calibration period for each area is summarised in Supplementary Table S3. As a baseline scenario, i.e. a scenario without any additional intervention, we projected future cases for 4 months since the latest date of observed data, using the most recent estimate of the transmission rate β_0_ and extrapolating the fitted model without any intervention.

In the scenario in which vaccination coverages are increased, the effect of additional vaccine uptake was assumed to work as a transition from the susceptible to the recovered state, i.e. the vaccine mode of action was assumed to be ‘all-or-nothing’ [33]). The transitioning proportion was calculated as S(t)(c_vac_–c_0_)VE, where S(t) is the susceptible proportion, VE is the vaccine effectiveness (assumed to be 60% [34]), and c_0 _and c_vac_ are the vaccination coverages before and after the additional vaccination. The baseline vaccination coverage c_0_ was set as 70% following the estimated coverage in Tokyo [29], and we examined the expected impacts of increased coverage by varying c_vac_ as 80% and 90%. The effect of NPIs was modelled as a reduction in the contact rate, and thus the transmission rate after implementing NPIs was formulated as β_NPIs _= (1–ф)β_0_, where ф is the reduced ratio of contact rate compared with the baseline. In the main analysis, the reduced ratio ф was set as 10%, and further reductions were examined in Supplementary Text. For both scenario analyses, we used the estimated baseline transmission rate β_0_ and its 2 SD ranges as the uncertainty ranges of projections.

## Results

### Wastewater data collected in study areas

While there was a large degree of noise in individual observations of virus concentrations in wastewater, smoothed wastewater data indicated that growing and declining trends roughly matched with those observed in case data, particularly in Kyoto city (Supplementary Figure S2). In Kanagawa, such growth trends were observed earlier in wastewater data than case data, while the City A exhibited the most noisy trends with an indication of an earlier increase in case data. As City A has the smallest population size among the examined study areas, this result indicated that wastewater data may become more noisy when the disease prevalence (or the absolute number of infected individuals) is low in the wastewater catchment area.

### Growth trends estimated with reported case and wastewater data

**Figure 1:**
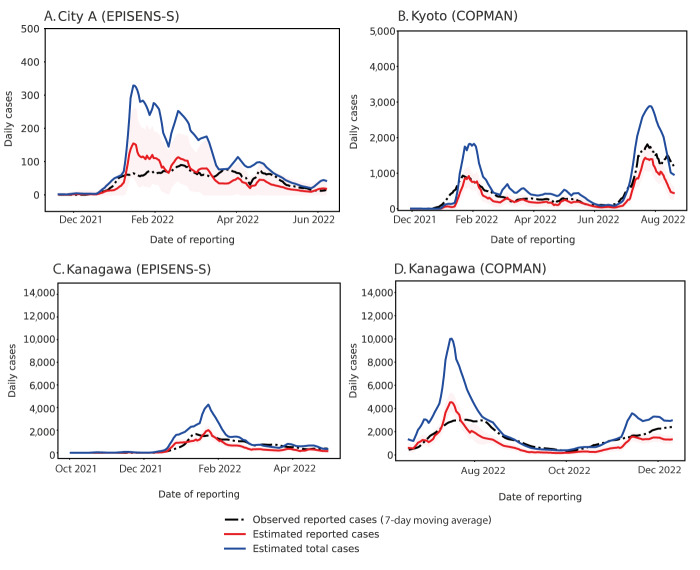
Estimated daily cases using only wastewater data in three areas in Japan, between November 2021−December 2022

The proposed modelling approach, using only wastewater data, described the epidemic trends in case data well at three study areas in Japan (Figure 1). Estimated parameters are listed in Supplementary Table S3. The estimated ranges of reported cases in Kyoto and Kanagawa matched with the observation during the initial growth of epidemic waves in January 2022, which demonstrates the compatibility between notification-based and wastewater-based surveillance. Observed reported cases were mostly within the estimated ranges of reported cases, and the large difference between the estimated total cases and the observed reported cases indicated that there may have been substantial under-reported cases around the peak of epidemic waves (Figure 1). Our additional analysis indicated that the selected parameters (i.e. assumed latent period, infectious period, and immunity duration) did not change the model fit substantially (provided in Supplementary Figure S3), supporting the robustness of our findings. By comparing different study areas, Figure 1 illustrates that the uncertainty in estimates increased for City A where the population size is the smallest among three study areas. The goodness of model fit, provided in panel C of Supplementary Figure S4, was slightly worse during the early period where the reported cases were limited in Kanagawa, suggesting that our approach would result in uncertain estimates when the disease incidence is low.

The black line indicates the observed daily reported cases, and the red line and shaded area represent the estimated daily reported cases with uncertainty bands of 2 standard deviations, respectively. The blue line corresponds to the estimated total cases, which was computed by incorporating under-reporting in the proposed modelling framework. COPMAN and EPISEN-S are different RNA extraction/detection methods, and the COPMAN method has a lower quantification limit of viral RNA.

**Figure 2:**
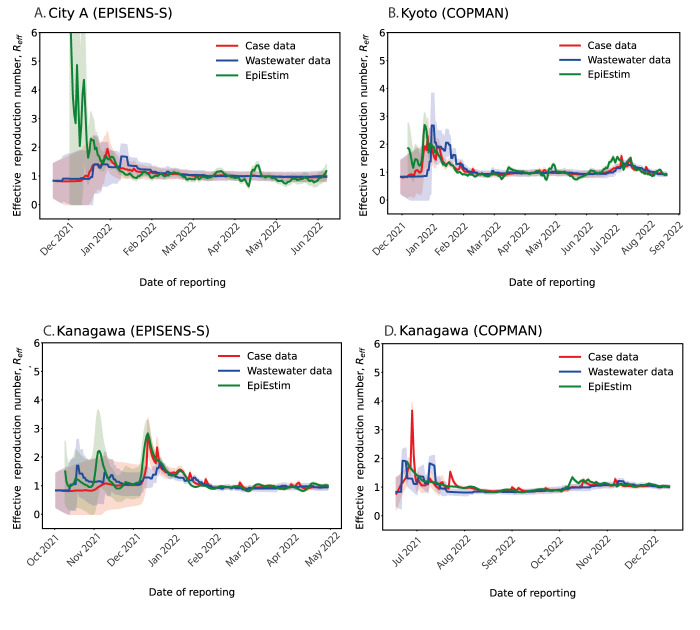
Estimated effective reproduction numbers using the proposed Kalman filter approach and the EpiEstim approach in three areas in Japan, between November 2021−December 2022

To further validate our findings, we compared two effective reproduction numbers, i.e. notification-based reproduction number R_eff_^N^ and wastewater-based reproduction number R_eff_^W^ (Figure 2, shown in blue and red). This analysis showed that the computed R_eff_^N^ and R_eff_^W^ were comparable throughout the study period, suggesting that our modelling approach using wastewater data can provide a reliable proxy for tracking epidemic trends. Besides, this was further supported by the result that the computed R_eff_^N^ and R_eff_^W^ with our approach were visually matched with the values of a standard EpiEstim approach (Figure 2, shown in green). In general, however, the estimated values of R_eff_^W^ produced smoother curves with respect to time compared with the estimated R_eff_^N^. This indicated that our approach with wastewater data alone may be less sensitive to abrupt changes in the epidemic, as the inherent noise in the data can hinder the identification of early signals.

Estimated effective reproduction numbers using the proposed Kalman filter approach with only notified case data (red) and only wastewater data (blue), and using the EpiEstim approach with only notified case data (green). Shaded areas represent uncertainty ranges of 2 standard deviations for the proposed Kalman filter approach (red and blue) and 95% credible intervals for EpiEstim approach (green).

### One-week ahead forecasting

**Figure 3:**
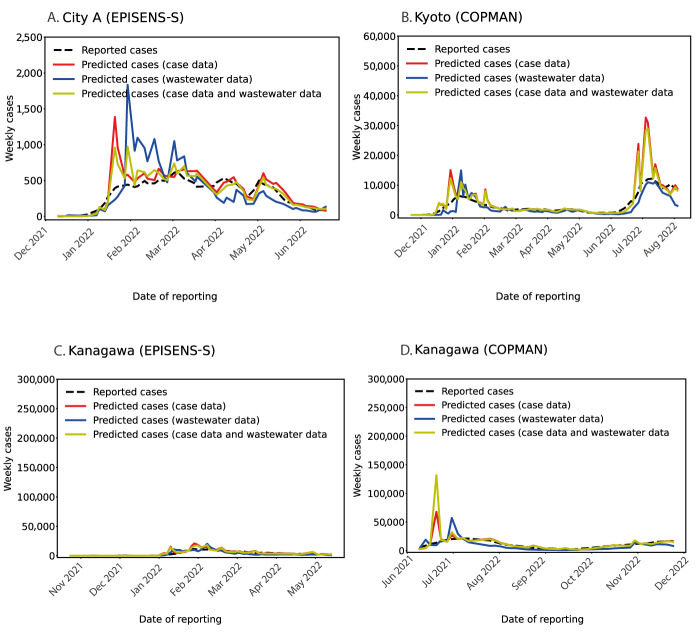
One-week ahead forecasting based on notified case data, wastewater data, or both in three areas in Japan, between November 2021−December 2022

We conducted 1-week ahead predictions of reported cases under three different conditions: using wastewater data only, case data only, and both wastewater and case data. To account for variations in observation frequency (two or three times per week), we aggregated daily case data over 1 week and compared the model predictions to the observed weekly number of cases. Figure 3 shows the 1-week ahead prediction of weekly cases across different study areas and RNA extraction/detection methods, and the examined three conditions did not show a significant difference in prediction abilities (Table). Interestingly, the model using both case and wastewater data did not necessarily show the best prediction performance, despite the utilisation of all available data for the prediction.

The simulated number of cases over 1 week was adaptively updated via the Kalman filter and was compared with the observed reported cases (black).

**Table:** Summary statistics for one-week ahead prediction errors in three areas in Japan, between November 2021−December 2022

Method		Municipalities			
		Kyoto city	City A	Kanagawa	
		COPMAN	EPISENS-S	COPMAN	EPISENS-S
MAE	Case data	1,813.7	92.0	2,980.7	1,271.0
	Wastewater data	1,388.5	173.6	4,840.6	1,599.6
	Case and wastewater data	1,561.3	84.9	4,679.1	1,277.0
RMSE	Case data	4,295.1	177.6	8,836.1	2,714.9
	Wastewater data	2,100.2	289.0	7,462.4	2,548.1
	Case and wastewater data	3,610.2	142.4	18,862.6	2,515.6

MAE: mean-absolute error; RMSE: root-mean-square error.

Lower values indicate smaller prediction errors. These metrics are calculated by comparing the predicted and actual numbers of cumulative new positive cases up to 1 week.

COPMAN and EPISENS-S are molecular methods to quantify RNA copies in wastewater (see details in Supplementary Materials).

### Scenario projections based on wastewater data

**Figure 4:**
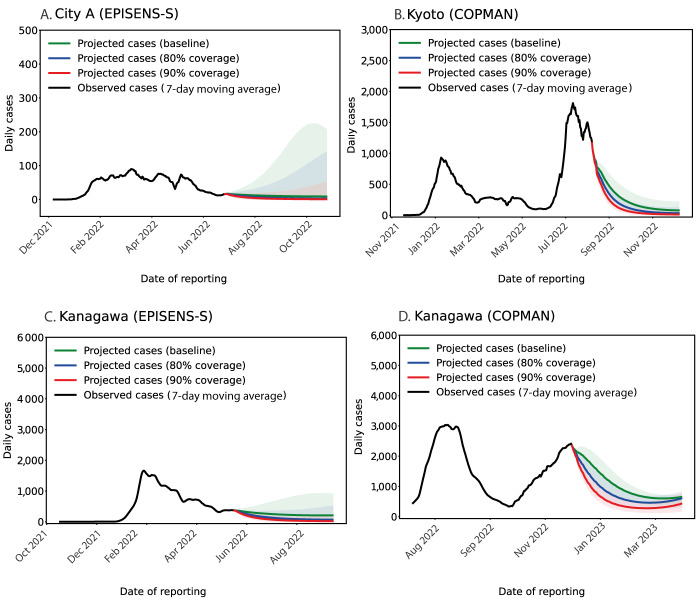
Model projected cases for increased vaccination scenarios in three areas in Japan, between November 2021−December 2022

**Figure 5:**
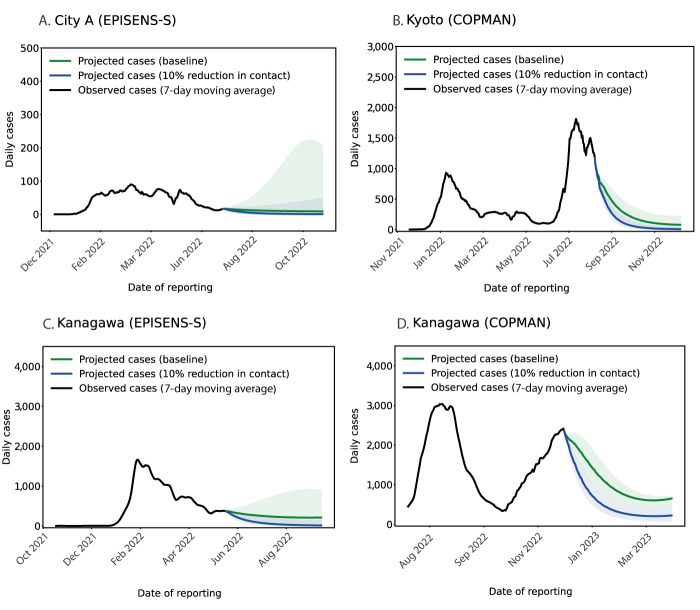
Model projected cases for a non-pharmaceutical intervention scenario in three areas in Japan, during 2022–2023

To demonstrate model-based projections, we visualised the potential impacts of two different strategies, i.e. increased vaccination coverage and NPIs, using the model calibrated with wastewater data alone (Figure 4 and 5). The forward simulations indicated that both strategies would expedite the decrease in daily cases when compared with the baseline scenario that imposes no additional interventions. While the projected baseline trajectories suggested an overall decreasing trend (green line in Figure 4 and 5), the uncertainty intervals in two study areas (Kanagawa and City A) indicated a possible increase in daily cases (green-shaded regions in Figure 4A and 4B and Figure 5A and B). The same trend in the baseline scenario can be more clearly seen in the projected cumulative cases, provided in Supplementary Figures S5 and S6). We also performed more stringent NPI scenarios, provided in Supplementary Figure S7–S10); those scenario analyses showed that an increase in vaccination by 10–20% or a reduction in the contact rate by ca 10% could alter the upper bound of the projected incidence into a declining trend in our simulation settings. Among the study areas, the largest reduction in projected cases was seen in Kanagawa during January–April 2023 where the incidence of cases was the highest (Figure 5D).

Vaccination coverages are set as 70% of the population for the baseline (green) and 80% (blue) and 90% (red) for scenarios with accelerated vaccine uptakes. Each shaded area represents uncertainty ranges of 2 standard deviations computed by the estimated variance of the baseline transmission rate.

Relative contact rates are set as 1 for the baseline (green) and 0.9 (blue) and for scenarios with reduced contact rates. Each ribbon represents uncertainty ranges of 2 standard deviations computed by the estimated variance of the baseline transmission rate.

## Discussion

In this study, we showed that wastewater can capture the underlying trend of circulating SARS-CoV-2 infections and presented how scenario analyses can be provided to guide a policy decision by the proposed modelling framework. Our modelling translated the observed growth trend in wastewater data into effective reproduction numbers, which were consistent with estimated values by notified case data. As an application example, we further conducted scenario-based modelling analyses to illustrate the impact of different types of interventions on the projected number of cases. This highlighted the benefit of incorporating wastewater data into the current scenario modelling framework, regardless of the virus quantification method, especially when reliable epidemiological data are not obtainable.

The transmission model used in this study provided a good description of wastewater data. While previous literature on wastewater surveillance often claimed that machine-learning based models could capture more complex dynamics [14,17], our mechanistic model with parsimonious parameterisations yielded comparable estimates of reproduction numbers for both case and wastewater data. The main strength of our modelling approach is that all parameters have biological or epidemiological interpretations, and thus the outputs can be used for further scenario analysis. The interpretability and explainability are essential for informing policymaking as well as for (external) validity checks, in cases where there is a drastic change in transmission dynamics, e.g. the emergence of new SARS-CoV-2 variants.

Real-time monitoring of effective reproduction numbers for SARS-CoV-2 via wastewater surveillance would be more effectively used if the notified case data are subject to substantial reporting delay or become less reliable (e.g. owing to changes in the reporting system). Effective reproduction numbers computed via case data are likely to capture the underlying growth trend of an epidemic, as long as the reporting rate is constant over the generation time. In our study period, there was no drastic change in reporting in Japan, and our analysis showed that wastewater-based reproduction numbers were consistent with notification-based reproduction numbers, suggesting that our approach can effectively monitor the epidemic trend via wastewater surveillance. Various methods have been proposed to compute effective reproduction numbers [32,35,36], and their limitations are widely discussed [30,37]. A common challenge is that those methods are prone to sudden changes in reporting system, e.g. case definition, testing policy, diagnostic capacity, etc. By contrast, wastewater surveillance is more robust to such transitions in the data collection process. Several approaches have been proposed to estimate effective reproduction numbers via wastewater data [18,20,21]. We proposed to extend the applicable range of this wastewater-based framework; reproduction numbers estimated by mechanistic modelling approaches, such as ours, would provide a coherent way to simulate possible trajectories of an epidemic by varying other parameters when the epidemiological situation is changing. This usability is important for the iterative policy-making process.

Using Japan as an example, we presented analyses by examining the impact of different intervention scenarios based on the proposed approach with the observed wastewater data. Our model projections showed that, in two study areas (City A and Kanagawa) where the daily incidence of COVID-19 was increasing, a 10–20% increase in the vaccination coverage or ca 10% reduction in the contact rate may be sufficient to turn the epidemic into a declining trend. These scenario analyses are useful to understand how much additional effort would be needed for controlling the disease on average. However, if more granular scenarios and strategic planning are required, e.g. targeted interventions by age, occupation, etc., additional epidemiological data would be essential, as wastewater data only captures an aggregated trend over the whole population in the catchment areas. In addition, the relationship between wastewater and case data may vary over time, and the calibration of models needs to be conducted together with the most recent data when available. Thus, wastewater surveillance is not the replacement of standard case monitoring, but rather it should be used as a complementary tool to support current epidemiological COVID-19 surveillance.

The present study provided insights for further improvements in wastewater surveillance and its applicability to scenario modelling. Our analysis suggested that the estimated growth trends via wastewater data were more consistent with case data when the prevalence was high and/or the population size covered by the sewage system was large. Conversely, when the prevalence is low, virus concentrations in wastewater would also become low and approach the detection limit, leading to uncertain RNA quantifications with larger variations. Although the sensitivity of molecular methods has been extensively discussed [8,38], the minimisation of variations in observations, e.g. experimental errors, variations in water sampling process, etc., is also the key to capture the underlying epidemic trends. While it is possible to incorporate unobserved variations with various modelling approaches, such as the one proposed in this study, the implementation of experimental and sampling systems with reduced errors, e.g. flow-proportional composite water sampling [13], would enhance the accuracy of wastewater surveillance and expediate more reliable scenario analysis.

Our scenario analysis should be interpreted with caution. Our formulation simplified the dynamics, and consequently various pathogen/host factors, e.g. age-dependent contact rates, infectivity and immune-escape effect by variant, seasonality, etc., were aggregated into the estimated parameters. Reporting delays from symptom onset to confirmation, or intrinsic delays from infection to detection of viruses in wastewater were summarised as a site-specific single parameter, i.e. mean shedding duration 1/γ in our model, and thus estimated values of this parameter should be carefully interpreted. In particular, the projected impacts of vaccination strategy may vary in practice, because of differences in the timing of vaccinations or differing waning rates by age. Although our aim was to illustrate the proposed framework by using collected data in Japan with minimal parameterisation, the model assumptions and possible extensions in the structure, such as age stratification, need to be considered when more data are available. For the best practice of scenario modelling, we should always accommodate alternative candidate models and should not rely on a single model, and scenario analysis needs to be adaptively updated.

## Conclusion

We have illustrated how wastewater data can be translated into intuitive epidemiological quantities such as total COVID-19 cases and reproduction numbers, and how we can use wastewater data as an alternative source of information for scenario modelling to inform future policy. The proposed framework with wastewater surveillance could be applicable to other viruses that have similar dynamics as SARS-CoV-2, and complements and maximises the benefit of clinical surveillance especially when reliable and timely epidemiological data are not available.

## Supplementary Data

1252000 Supplement

## References

[1] Ainslie KEC Backer JA de Boer PT van Hoek AJ Klinkenberg D Korthals Altes H A scenario modelling analysis to anticipate the impact of COVID-19 vaccination in adolescents and children on disease outcomes in the Netherlands, summer 2021. Euro Surveill. 2022;27(44):2101090. 10.2807/1560-7917.ES.2022.27.44.2101090 36330824 PMC9635025

[2] Keeling MJ Dyson L Tildesley MJ Hill EM Moore S. Comparison of the 2021 COVID-19 roadmap projections against public health data in England. Nat Commun. 2022;13(1):4924. 10.1038/s41467-022-31991-0 35995764 PMC9395530

[3] COVID 19 Scenario Modeling Hub. COVID 19 scenario modeling hub. [Accessed: 5 Mar 2023]. Available from: https://covid19scenariomodelinghub.org

[4] European Covid-19 Scenario Hub. European Covid-19 Scenario Hub. [Accessed: 5 Mar 2023]. Available from: https://covid19scenariohub.eu

[5] Leung K Lau EHY Wong CKH Leung GM Wu JT. Estimating the transmission dynamics of Omicron in Beijing, November to December 2022. BioRxiv 2022. 10.1101/2022.12.15.22283522 36638825

[6] Klous G, McDonald S, de Boer P, van Hoek AJ, Franz E, van Rooijen M. Staat van Infectieziekten in Nederland, 2021. RIVM rapport 2022-0141. [State of Infectious Diseases in the Netherlands, 2021]. Bilthoven: Rijksinstituut voor Volksgezondheid en Milieu (RIVM); 2022. Available from:

[7] Naughton CC Roman FA Jr Alvarado AGF Tariqi AQ Deeming MA Kadonsky KF Show us the data: global COVID-19 wastewater monitoring efforts, equity, and gaps. FEMS Microbes. 2023;4:xtad003. 10.1093/femsmc/xtad003 37333436 PMC10117741

[8] Hill V Githinji G Vogels CBF Bento AI Chaguza C Carrington CVF Toward a global virus genomic surveillance network. Cell Host Microbe. 2023;31(6):861-73. 10.1016/j.chom.2023.03.003 36921604 PMC9986120

[9] Kitajima M Ahmed W Bibby K Carducci A Gerba CP Hamilton KA SARS-CoV-2 in wastewater: State of the knowledge and research needs. Sci Total Environ. 2020;739:139076. 10.1016/j.scitotenv.2020.139076 32758929 PMC7191289

[10] Wolfe MK Yu AT Duong D Rane MS Hughes B Chan-Herur V Use of wastewater for mpox outbreak surveillance in California. N Engl J Med. 2023;388(6):570-2. 10.1056/NEJMc2213882 36652340

[11] Ryerson AB Lang D Alazawi MA Neyra M Hill DT St George K Wastewater testing and detection of poliovirus type 2 genetically linked to virus isolated from a paralytic polio case - New York, March 9-October 11, 2022. MMWR Morb Mortal Wkly Rep. 2022;71(44):1418-24. 10.15585/mmwr.mm7144e2 36327157 PMC9639435

[12] Wastewater SPHERE. Wastewater SPHERE (SARS Public Health Environmental REsponse). [Accessed: 23 Mar 2023]. Available from: https://sphere.waterpathogens.org

[13] Ahmed W Bivins A Bertsch PM Bibby K Choi PM Farkas K Surveillance of SARS-CoV-2 RNA in wastewater: Methods optimisation and quality control are crucial for generating reliable public health information. Curr Opin Environ Sci Health. 2020;17:82-93. 10.1016/j.coesh.2020.09.003 33052320 PMC7544017

[14] Jiang G Wu J Weidhaas J Li X Chen Y Mueller J Artificial neural network-based estimation of COVID-19 case numbers and effective reproduction rate using wastewater-based epidemiology. Water Res. 2022;218:118451. 10.1016/j.watres.2022.118451 35447417 PMC9006161

[15] Morvan M Jacomo AL Souque C Wade MJ Hoffmann T Pouwels K An analysis of 45 large-scale wastewater sites in England to estimate SARS-CoV-2 community prevalence. Nat Commun. 2022;13(1):4313. 10.1038/s41467-022-31753-y 35879277 PMC9312315

[16] van Boven M Hetebrij WA Swart AM Nagelkerke E van der Beek RF Stouten S Modelling patterns of SARS-CoV-2 circulation in the Netherlands, August 2020-February 2022, revealed by a nationwide sewage surveillance program. MedRxiv 2022. . 10.1101/2022.05.25.22275569 PMC10288829 37347416

[17] Li X Kulandaivelu J Zhang S Shi J Sivakumar M Mueller J Data-driven estimation of COVID-19 community prevalence through wastewater-based epidemiology. Sci Total Environ. 2021;789:147947. 10.1016/j.scitotenv.2021.147947 34051491 PMC8141262

[18] Proverbio D Kemp F Magni S Ogorzaly L Cauchie H-M Gonçalves J Model-based assessment of COVID-19 epidemic dynamics by wastewater analysis. Sci Total Environ. 2022;827:154235. 10.1016/j.scitotenv.2022.154235 35245552 PMC8886713

[19] Phan T Brozak S Pell B Gitter A Mena KD Kuang Y A simple SEIR-V model to estimate COVID-19 prevalence and predict SARS-CoV-2 transmission using wastewater-based surveillance data. MedRxiv 2022 . 10.1101/2022.07.17.22277721 PMC9547654 36220466

[20] Nourbakhsh S Fazil A Li M Mangat CS Peterson SW Daigle J A wastewater-based epidemic model for SARS-CoV-2 with application to three Canadian cities. Epidemics. 2022;39:100560. 10.1016/j.epidem.2022.100560 35462206 PMC8993419

[21] Huisman JS Scire J Caduff L Fernandez-Cassi X Ganesanandamoorthy P Kull A Wastewater-based estimation of the effective reproductive number of SARS-CoV-2. Environ Health Perspect. 2022;130(5):57011. 10.1289/EHP10050 35617001 PMC9135136

[22] Ando H Iwamoto R Kobayashi H Okabe S Kitajima M. The Efficient and Practical virus Identification System with ENhanced Sensitivity for Solids (EPISENS-S): A rapid and cost-effective SARS-CoV-2 RNA detection method for routine wastewater surveillance. Sci Total Environ. 2022;843:157101. 10.1016/j.scitotenv.2022.157101 35952875 PMC9357991

[23] Adachi Katayama Y Hayase S Ando Y Kuroita T Okada K Iwamoto R COPMAN: A novel high-throughput and highly sensitive method to detect viral nucleic acids including SARS-CoV-2 RNA in wastewater. Sci Total Environ. 2023;856(Pt 1):158966. 10.1016/j.scitotenv.2022.158966 36162583 PMC9502438

[24] Yamayoshi S Yasuhara A Ito M Akasaka O Nakamura M Nakachi I Antibody titers against SARS-CoV-2 decline, but do not disappear for several months. EClinicalMedicine. 2021;32:100734. 10.1016/j.eclinm.2021.100734 33589882 PMC7877219

[25] Puhach O Meyer B Eckerle I. SARS-CoV-2 viral load and shedding kinetics. Nat Rev Microbiol. 2023;21(3):147-61. 36460930 10.1038/s41579-022-00822-w PMC9716513

[26] Backer JA Eggink D Andeweg SP Veldhuijzen IK van Maarseveen N Vermaas K Shorter serial intervals in SARS-CoV-2 cases with Omicron BA.1 variant compared with Delta variant, the Netherlands, 13 to 26 December 2021. Euro Surveill. 2022;27(6):2200042. 10.2807/1560-7917.ES.2022.27.6.2200042 35144721 PMC8832521

[27] King AA Ionides EL Pascual M Bouma MJ. Inapparent infections and cholera dynamics. Nature. 2008;454(7206):877-80. 10.1038/nature07084 18704085

[28] Shaman J Karspeck A Yang W Tamerius J Lipsitch M. Real-time influenza forecasts during the 2012-2013 season. Nat Commun. 2013;4(1):2837. 10.1038/ncomms3837 24302074 PMC3873365

[29] Tokyo Metropolitan Government. Tokyo Metropolitan Government COVID-19 Information Website. Tokyo Metropolitan Government COVID-19 Information Website. [Accessed: 23 Mar 2023]. Available from: https://www.hokeniryo.metro.tokyo.lg.jp/kansen/corona_portal/info/covid19_opendata.html

[30] Gostic KM McGough L Baskerville EB Abbott S Joshi K Tedijanto C Practical considerations for measuring the effective reproductive number, Rt. PLOS Comput Biol. 2020;16(12):e1008409. 10.1371/journal.pcbi.1008409 33301457 PMC7728287

[31] Fraser C. Estimating individual and household reproduction numbers in an emerging epidemic. PLoS One. 2007;2(8):e758. 10.1371/journal.pone.0000758 17712406 PMC1950082

[32] Cori A Ferguson NM Fraser C Cauchemez S. A new framework and software to estimate time-varying reproduction numbers during epidemics. Am J Epidemiol. 2013;178(9):1505-12. 10.1093/aje/kwt133 24043437 PMC3816335

[33] Halloran ME. Longini Jr. IM, Struchiner CJ. Design and Analysis of Vaccine Studies. Springer, New York, NY; 2010.

[34] Braeye T Catteau L Brondeel R van Loenhout JAF Proesmans K Cornelissen L Vaccine effectiveness against transmission of alpha, delta and omicron SARS-COV-2-infection, Belgian contact tracing, 2021-2022. Vaccine. 2023;41(20):3292-300. 10.1016/j.vaccine.2023.03.069 37085456 PMC10073587

[35] Wallinga J Teunis P. Different epidemic curves for severe acute respiratory syndrome reveal similar impacts of control measures. Am J Epidemiol. 2004;160(6):509-16. 10.1093/aje/kwh255 15353409 PMC7110200

[36] Parag KV. Improved estimation of time-varying reproduction numbers at low case incidence and between epidemic waves. PLOS Comput Biol. 2021;17(9):e1009347. 10.1371/journal.pcbi.1009347 34492011 PMC8448340

[37] Thompson RN Stockwin JE van Gaalen RD Polonsky JA Kamvar ZN Demarsh PA Improved inference of time-varying reproduction numbers during infectious disease outbreaks. Epidemics. 2019;29:100356. 10.1016/j.epidem.2019.100356 31624039 PMC7105007

[38] Hewitt J Trowsdale S Armstrong BA Chapman JR Carter KM Croucher DM Sensitivity of wastewater-based epidemiology for detection of SARS-CoV-2 RNA in a low prevalence setting. Water Res. 2022;211:118032. 10.1016/j.watres.2021.118032 35042077 PMC8720482

