# Supplementary material for: Wastewater-based reproduction numbers and projections of COVID-19 cases in three areas in Japan, November 2021 to December 2022
Source: Euro Surveill. 2024 Feb 22;29(8):2300277. doi: 10.2807/1560-7917.ES.2024.29.8.2300277 (PMC10899819; doi:10.2807/1560-7917.ES.2024.29.8.2300277)
Supplement: Supplement [file 23-00277_MIURA_Supplement.pdf]

"This supplementary material is hosted by Eurosurveillance as supporting information alongside the article Wastewater-based reproduction numbers and projections of COVID-19 cases in multiple cities in Japan, 2022 on behalf of the authors who remain responsible for the accuracy and appropriateness of the content. The same standards for ethics, copyright, attributions and permissions as for the article apply. Eurosurveillance is not responsible for the maintenance of any links or email addresses provided therein."

Supplementary Materials for

Wastewater-based reproduction numbers and projections of COVID-19 cases in three areas in Japan, November 2021 to December 2022

Table of contents:

Supplementary Text

Wastewater sampling
Molecular analysis of wastewater samples
Parameter estimation and sequential update
State update method with extended Kalman filter
Estimation of region-specific parameters
Computation of reporting rate Alternative vaccination scenario

Supplementary figures and tables

Figure S1. Schematic illustration of model structure
Figure S2. Collected wastewater and case data
Figure S3. Sensitivity analysis and summarized error metrics
Figure S4. Summarized error metrics of model fit at different time points
Figure S5. Projected cumulative cases in vaccination scenarios
Figure S6. Projected cumulative cases in non-pharmaceutical intervention scenarios
Figure S7. Additional analysis for more stringent NPIs scenario (15% reduction in contact)
Figure S8. Additional analysis for more stringent NPIs scenario (20% reduction in contact)
Figure S9. Additional analysis for more stringent NPIs scenario (25% reduction in contact)
Figure S10. Additional analysis for more stringent NPIs scenario (30% reduction in contact)

Table S1. Data source
Table S2. List of input parameters
Table S3. List of estimated parameters
Table S4. List of initial parameters.

Supplementary Text

Wastewater sampling

Influent wastewater samples were collected from wastewater treatment plants in three study areas in Japan (see **Methods** and **Table S1**). The wastewater samples were collected in sterile plastic bottles via grab sampling and immediately transported to the laboratory. Grab samplings were conducted mostly between 9 a.m. to 12 p.m. when the fecal concentration is likely to reach its peak at sampling points. The samples were processed with the concentration method described below on the day of sample collection. Sampling frequencies were two or three times per week but this number of samples varied due to national holidays and the operation capacity of wastewater treatment plants (**Table S1**).

Molecular analysis of wastewater samples

The collected wastewater samples were analyzed by EPISENS-S [1] and COPMAN [2] to quantify the SARS-CoV-2 and PMMoV RNA concentration in wastewater. Briefly, for EPISENS-S, total RNA was extracted from suspended solid formed via low-speed centrifugation at 3,000 g for 10 mins in the 40 mL sewage samples, using the RNeasy PowerMicrobiome kit on a QIAcube system (Qiagen, Hilden, Germany) to obtain a final RNA extracted volume of 50 µL. One-step RT-preamplification was performed using 13.5 µL of the RNA extract and the CDC N1 forward and reverse (2019-nCoV_N1-F and 2019-nCoV_N1-R) and PMMoV reverse (PMMV_RP1) primers (Table S1, S2 in the Supplementary Material of [1]) with the iScript™ Explore One-Step RT and PreAmp Kit (Bio-Rad Laboratories, Hercules, CA, USA). Thermal cycling conditions for RT-preamplification were as follows; 25℃ for 5 mins, 45℃ for 60 mins, 95℃ for 3 mins followed by 10 cycles of 95℃ for 15 s and 55℃ for 4 mins. qPCR was performed with QuantiTect Probe PCR Master Mix (Qiagen, Hilden, Germany) in a total reaction volume of 25 µL containing 2.5 µL of pre-amplified products, which was performed using the primers and probe for SARS-CoV-2 (CDC N1) or PMMoV with a final concentration of 400 nM and 300 nM each, respectively (Table S1 and S2 in the Supplementary Material of [1]). Thermal cycling conditions for qPCR were as follows; 50℃ for 2 mins, 95℃ for 10 mins followed by 45 cycles of 95℃ for 3 s and 55℃ for 32 s. qPCR reactions were completed on ABI 7500 Real-Time qPCR system (Applied Biosystems), and the threshold value of relative fluorescent intensity (ΔRn) was adjusted to be 0.2.

For COPMAN, viruses were coagulated with the addition of 1 µL of polyaluminum chloride (PAC) followed by vigorous shaking for 30 times, and subsequent gentle shaking at 80–120 rpm for 10 min at 4 °C. The samples were then centrifuged at 3000 ×g for 10 min, and the supernatant was discarded. The samples were centrifuged again at 3000 ×g for 3 min and the remaining liquids were removed by pipetting. The debris was then transferred to a 1.5-mL tube and lysed with 250-µL SDS-based lysis buffer and digested by 14.25-µL proteinase K solution at 56 °C for 10 min. Crude RNA of 200 µL was extracted from the samples with phenol/chloroform/isoamyl alcohol (25:24:1), which was then purified with carboxyl-modified magnetic beads, to obtain a final RNA extract volume of 50 µL. An aliquot (2 µg or 13 µL) of the magnetic bead-purified total RNA was subjected to cDNA synthesis using the Reliance Select cDNA synthesis kit (Bio-Rad Laboratories) under the following conditions: 50 °C for 60 min, 95 °C for 1 min in 20-µL reaction mix with 2 pmol each of reverse primers of SARS-CoV-2, RSV, and PMMoV. The resultant cDNAs of SARS-CoV-2 and RSV were pre-amplified for 10 cycles by the Biotaq HS (Bioline Reagents Ltd., London, UK) under the following conditions: 95 °C for 10 min, and 10 cycles of 95 °C for 15 s, 55 °C for 15 s, and 72 °C for 30 s, in 30-µL volume reaction mix containing 9 pmol each of forward and reverse primers. PMMoV cDNA was not preamplified because PMMoV RNA usually exists in wastewater with high amounts. Finally, viral RNA was quantified from 2.5 µL of the preamp product for SARS-CoV-2 and RSV, and 2.5 µL of cDNA for PMMoV by qPCR using the TaqMan Environmental Master Mix 2.0 (Thermo Fisher Scientific) under the following conditions: 95 °C for 10 min, and 45 cycles of 95 °C for 15 s and 60 °C for 30 s, in 20-µL singleplex reaction mix containing 10 pmol each of reverse and forward primers and 7.5 pmol of TaqMan probe.

Parameter estimation and sequential update

Our model consists of pre-defined parameters (**Table S2**) and free three parameters to be estimated. Two region-specific parameters, the shedding duration $1/\gamma$ and the scaling parameter ν, were estimated by fitting the model to both notified case data and wastewater data, using initial weeks as calibration period (see calibration periods for each study area in **Table S3**). These estimated parameters are constant over the study period. Another time-varying parameter, the transmission rate $\beta(t)$, was estimated by fitting the model to either notified case data or wastewater data (or both). This estimate was sequentially updated at each time point.

State update method with extended Kalman filter

We update the state vector $\boldsymbol{x}(t) = [S, E, I, R, A, \beta](t)$ and the error covariance matrix $\boldsymbol{P}$ using the extended Kalman filter, following the method of [3] that uses the general extended Kalman filter [4]. The model described in the main text (in the section of Stochastic SEIRS model and observation process) is used as the state update function, and the state update is performed using the observation matrix $\boldsymbol{C}_t$ (case data, or wastewater data, or both), the state noise $\boldsymbol{Q}$, the measurement error covariance $\boldsymbol{U}(t)$, and the Jacobian $\boldsymbol{J}_f$ of the state update function [3,4]. The time step is set to Δt=1/10 days.

Estimation of region-specific parameters

The mean duration of virus shedding $1/\gamma$ and scaling parameter ν are region-specific parameters to be estimated. In our analysis, we used the estimated region-specific parameters for the whole study period assuming they are constant over

time. If new strains emerge or wastewater sampling methods are changed, it is possible (and advisable) to update them by re-estimating these values with newer calibration data. The calibration periods for each study area are summarized in **Table S3**.

We used both case and wastewater data during this calibration, and searched the optimal values of γ and ν by minimizing the squared error between observations and model-predictions [3]. For computational efficiency, we set the constraints on the range of γ and ν as [0.2, 4] and [0.4, 1], respectively.

Computation of reporting rate

The reporting rate (μ_t) was computed by adjusting the day-of-week effect, holiday effect, and the overall ascertainment rate (i.e., the proportion of reported cases over all infected individuals), following the method of [3]. The overall ascertainment rate $1/\eta$ is assumed to be 0.44 based on the value estimated by previous study in Japan (in the code, the value is rounded and set as $\eta = 2.2$) [5]. To calculate the reporting proportion on each day of the week, we used first five weeks of the data ($t \leq 35$), and then update the reporting rate for each day sequentially:

$$\widetilde{\mu_t} = \begin{cases} \dfrac{35}{5} \dfrac{\sum_{j=0}^{4} y_C(mod(t-1,7)+1+7j)}{\sum_{s=1}^{35} y_C(s)} & for\ t \leq 35, \\ \dfrac{21}{3} \dfrac{y_C(t-7)+y_C(t-14)+y_C(t-21)}{\sum_{s=t-20}^{t} y_C(s)} & for\ t > 35 \end{cases}$$

Subsequently, these values are normalized by the weekly moving average, formulated as:

$$\mu_t = \frac{7\,\widetilde{\mu_t}}{\eta \sum_{s=t-6}^{t} \widetilde{\mu_s}}$$

The holiday effect was further incorporated by reducing the average reporting rate of the week by 75%, which is determined by the observed maximum change in testing rates in Tokyo during December 2022 [6].

Alternative vaccination scenario

In additional analysis where vaccination coverages are increased, the effect of vaccines was assumed to work as a proportional reduction in transmission rates (i.e., the vaccine mode of action was assumed to be "leaky" [7]), and the transmission rate after additional vaccination is:

$$\beta_{vac} = \frac{(1 - VE * c_{vac})}{(1 - VE * c_0)} \beta_0$$

where VE is the vaccine efficacy (assumed to be 60%) and c_0 and c_{vac} are the vaccination coverages before and after the additional vaccination, respectively. The baseline vaccination coverage c_0 was set as 70%, and we examined the expected impacts of increased coverage by varying c_{vac} as 0.8 and 0.9. The results of projections are shown in **Figure-S3**.

Reference

[1] Ando H, Iwamoto R, Kobayashi H, Okabe S, Kitajima M. The Efficient and Practical virus Identification System with ENhanced Sensitivity for Solids (EPISENS-S): A rapid and cost-effective SARS-CoV-2 RNA detection method for routine wastewater surveillance. Sci Total Environ 2022;843:157101.

[2] Adachi Katayama Y, Hayase S, Ando Y, Kuroita T, Okada K, Iwamoto R, et al. COPMAN: A novel high-throughput and highly sensitive method to detect viral nucleic acids including SARS-CoV-2 RNA in wastewater. Sci Total Environ 2023;856:158966.

[3] Proverbio D, Kemp F, Magni S, Ogorzaly L, Cauchie H-M, Gonçalves J, et al. Model-based assessment of COVID-19 epidemic dynamics by wastewater analysis. Sci Total Environ 2022;827:154235.

[4] Thrun S, Burgard W, Fox D. Probabilistic Robotics. MIT Press; 2005.

[5] Omori R, Mizumoto K, Nishiura H. Ascertainment rate of novel coronavirus disease (COVID-19) in Japan. Int J Infect Dis 2020;96:673–5.

[6] Tokyo Metropolitan Government. Tokyo Metropolitan Government COVID-19 Information Website. Tokyo Metropolitan Government COVID-19 Information Website n.d. https://stopcovid19.metro.tokyo.lg.jp/en/monitoring (accessed March 23, 2023).

[7] Halloran ME, LonginiJr. IM, Struchiner CJ. Design and Analysis of Vaccine Studies. Springer, New York, NY; 2010.

[8] Puhach O, Meyer B, Eckerle I. SARS-CoV-2 viral load and shedding kinetics. Nat Rev Microbiol 2023;21:147–61.

[9] Backer JA, Eggink D, Andeweg SP, Veldhuijzen IK, van Maarseveen N, Vermaas K, et al. Shorter serial intervals in SARS-CoV-2 cases with Omicron BA.1 variant compared with Delta variant, the Netherlands, 13 to 26 December 2021. Eurosurveillance 2022;27:2200042.

[10] Yamayoshi S, Yasuhara A, Ito M, Akasaka O, Nakamura M, Nakachi I, et al. Antibody titers against SARS-CoV-2 decline, but do not disappear for several months. EClinicalMedicine 2021;32:100734.

[11] Ren Z, Nishimura M, Tjan LH, Furukawa K, Kurahashi Y, Sutandhio S, et al. Large-scale serosurveillance of COVID-19 in Japan: Acquisition of neutralizing antibodies for Delta but not for Omicron and requirement of booster vaccination to overcome the Omicron's outbreak. PLoS One 2022;17:e0266270.

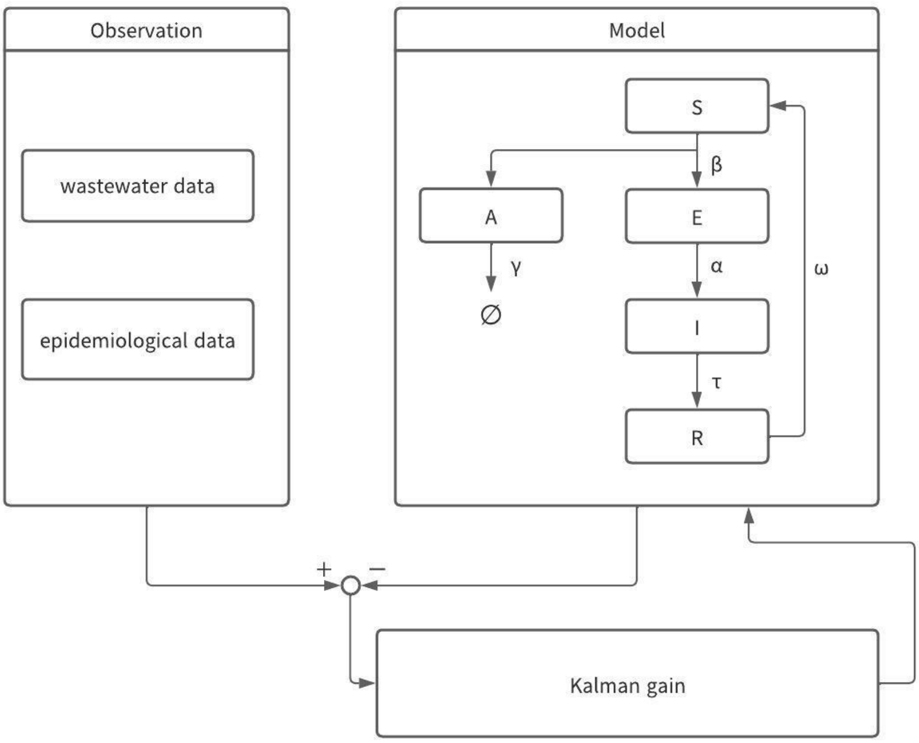

Figure S1. Schematic illustration of model structure.

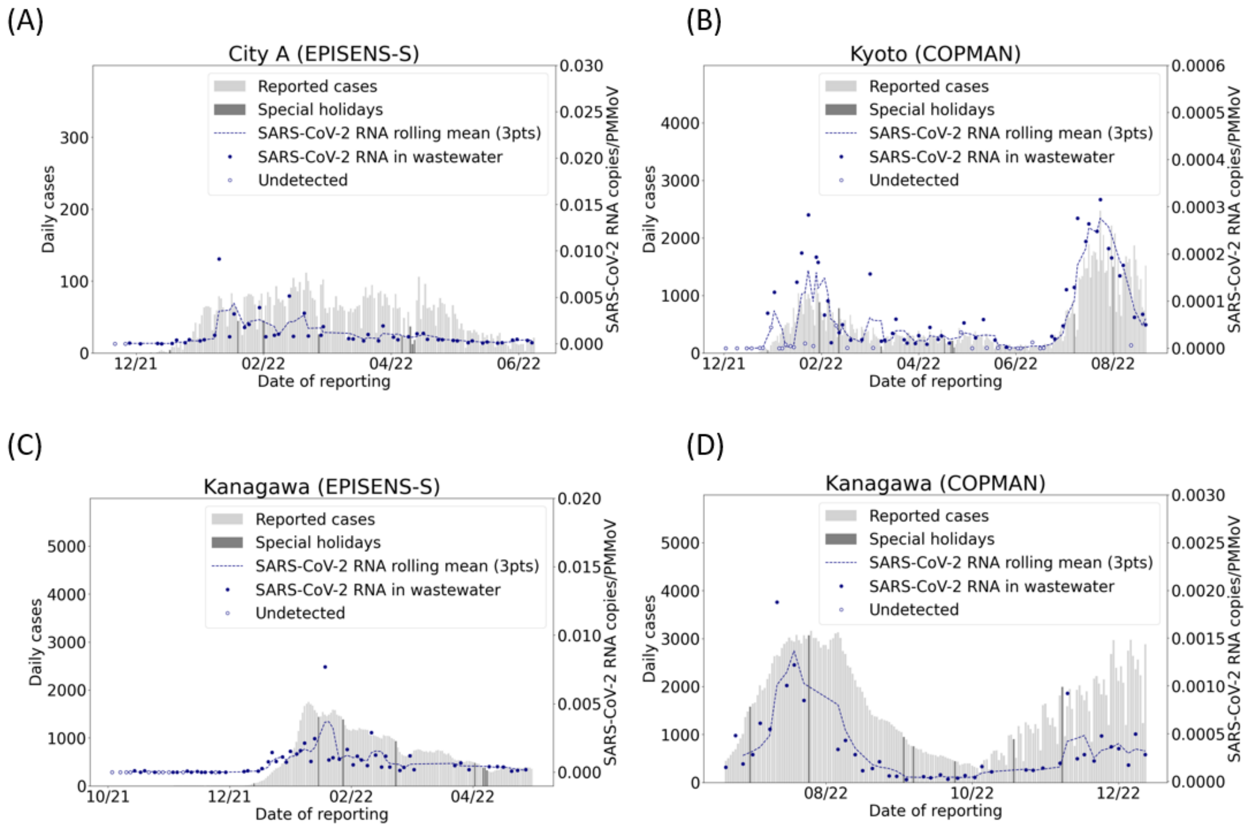

Figure S2. Collected wastewater and daily confirmed case data for different sites. Bars represent the daily number of reported cases, and darker bars indicate the cases reported on special holidays. Dot plots are SARS-CoV-2 RNA concentrations, and its rolling mean over three observed data points is shown as a blue line. White dots represent data points below the detection limit of RNA quantification methods.

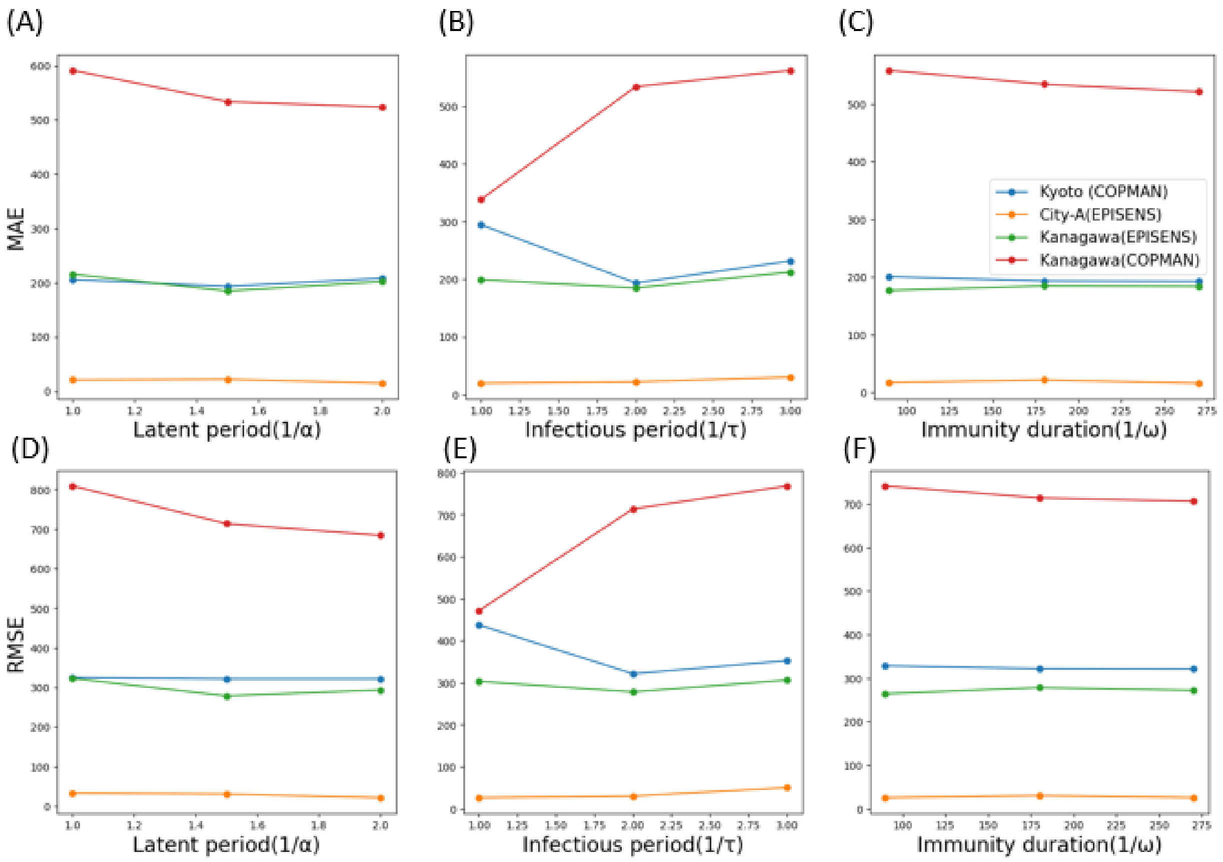

Figure S3. Sensitivity analysis and summarized error metrics. Colours indicate different study areas. X-axis is the varied parameter where other parameters are fixed as the same parameters in the main analysis. Errors between observations and model-predicted values are measured by mean-squared-error (MAE) (panel A, B, and C) and root-mean-squared-error (RMSE) (panel D, E, and F).

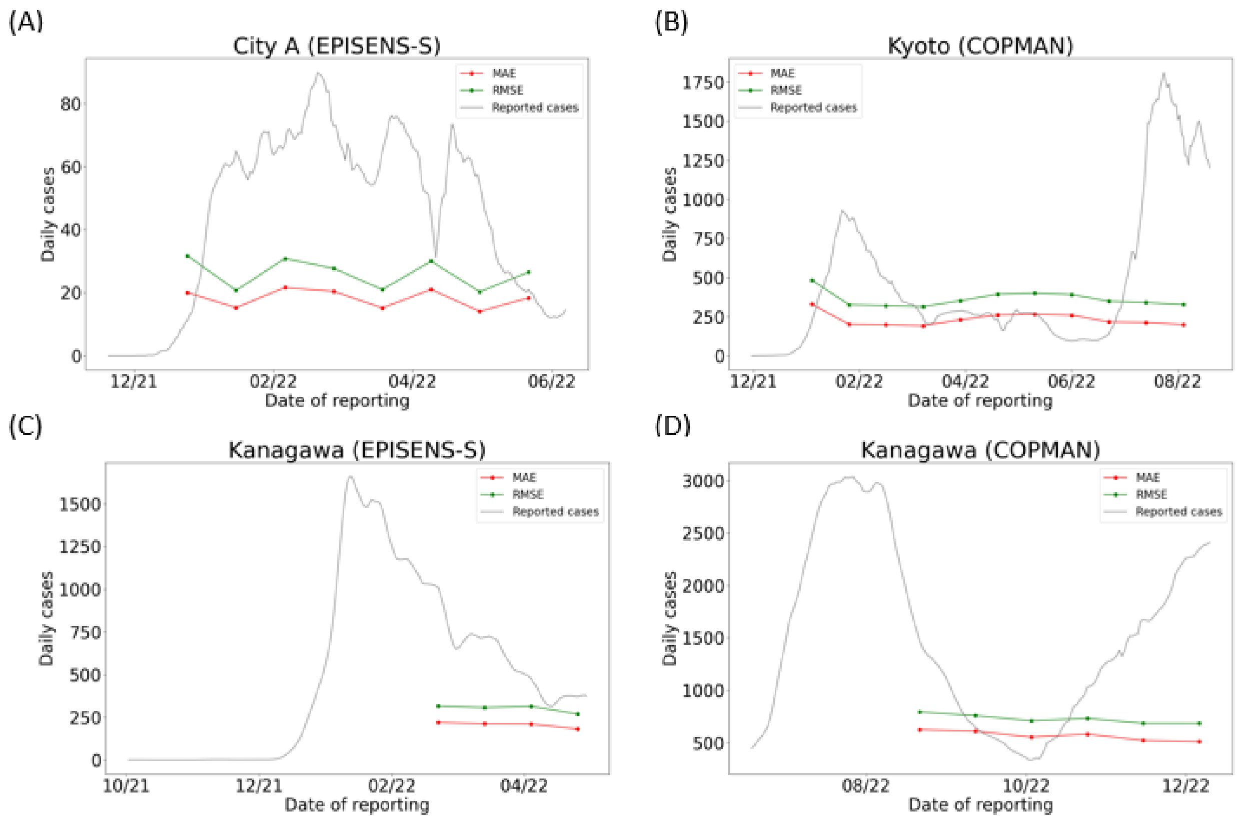

Figure S4. Summarized error metrics of model fit at different time points. Black lines indicate observed cases, and blue and red lines show summary statistics of model fits. Errors between observations and model-predicted values are measured by mean-squared-error (MAE) (red) and root-mean-squared-error (RMSE) (green). Both MAE and RMSE were calculated using the most recent (past) three weeks from the plotted time point.

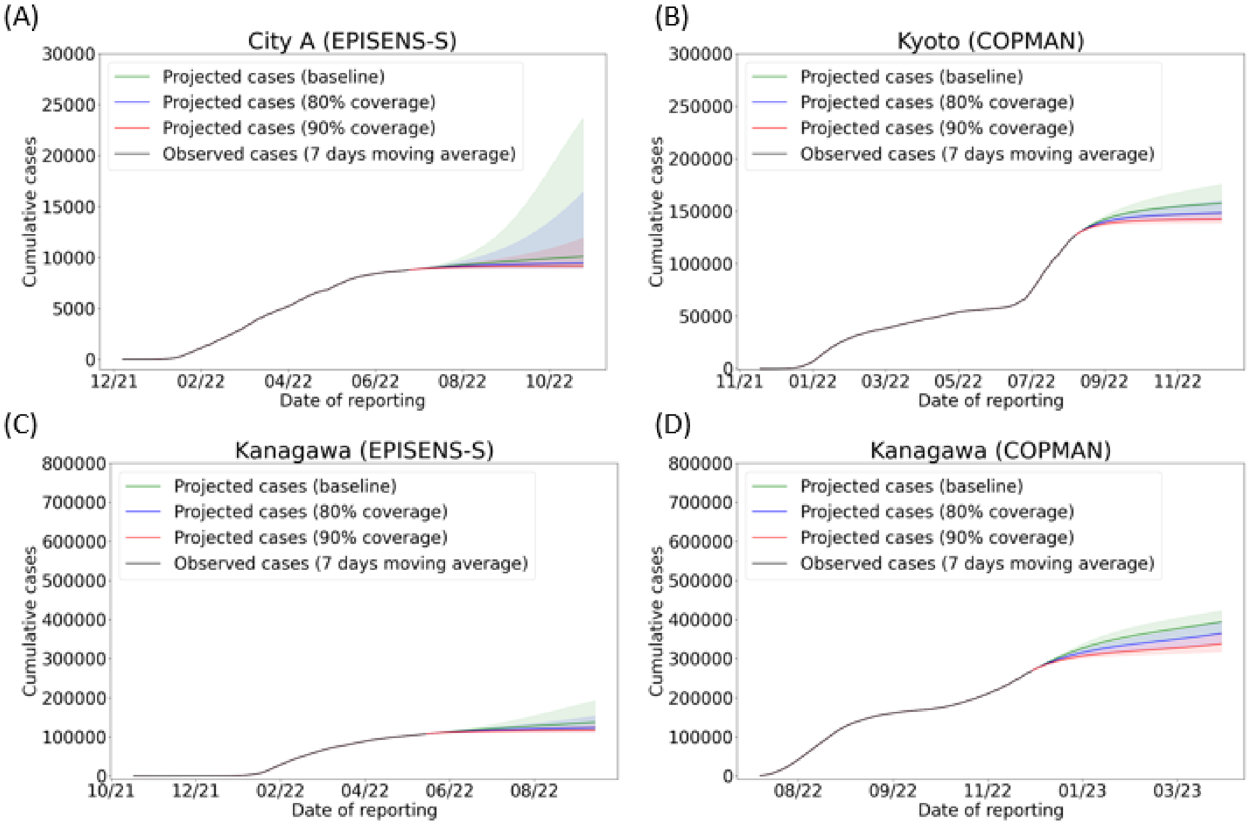

Figure S5. Projected cumulative cases in vaccination scenarios. Vaccine effect is assumed to reduce the baseline transmission rate proportionally (i.e., “leaky” effect). Vaccination coverages are set as 70% of the population for the baseline (green) and 80% (blue) and 90% (red) for scenarios with accelerated vaccine uptakes. Each ribbon represents uncertainty ranges of 2 standard deviations (SD) computed by the estimated variance of the baseline transmission rate.

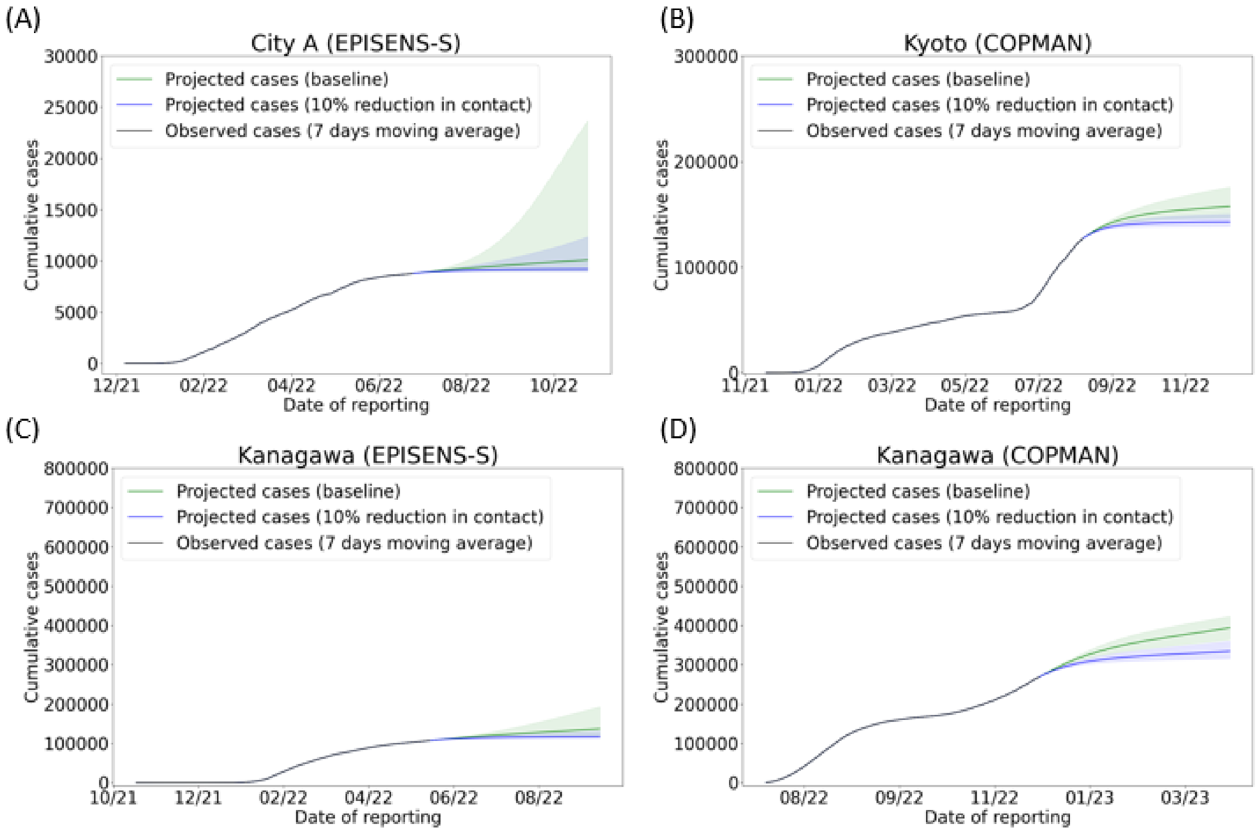

Figure S6. Projected cumulative cases in non-pharmaceutical intervention scenarios. Relative contact rates are set as 1 for the baseline (green) and 0.9 (blue) and for scenarios with reduced contact rates. Each ribbon represents uncertainty ranges of 2 standard deviations (SD) computed by the estimated variance of the baseline transmission rate.

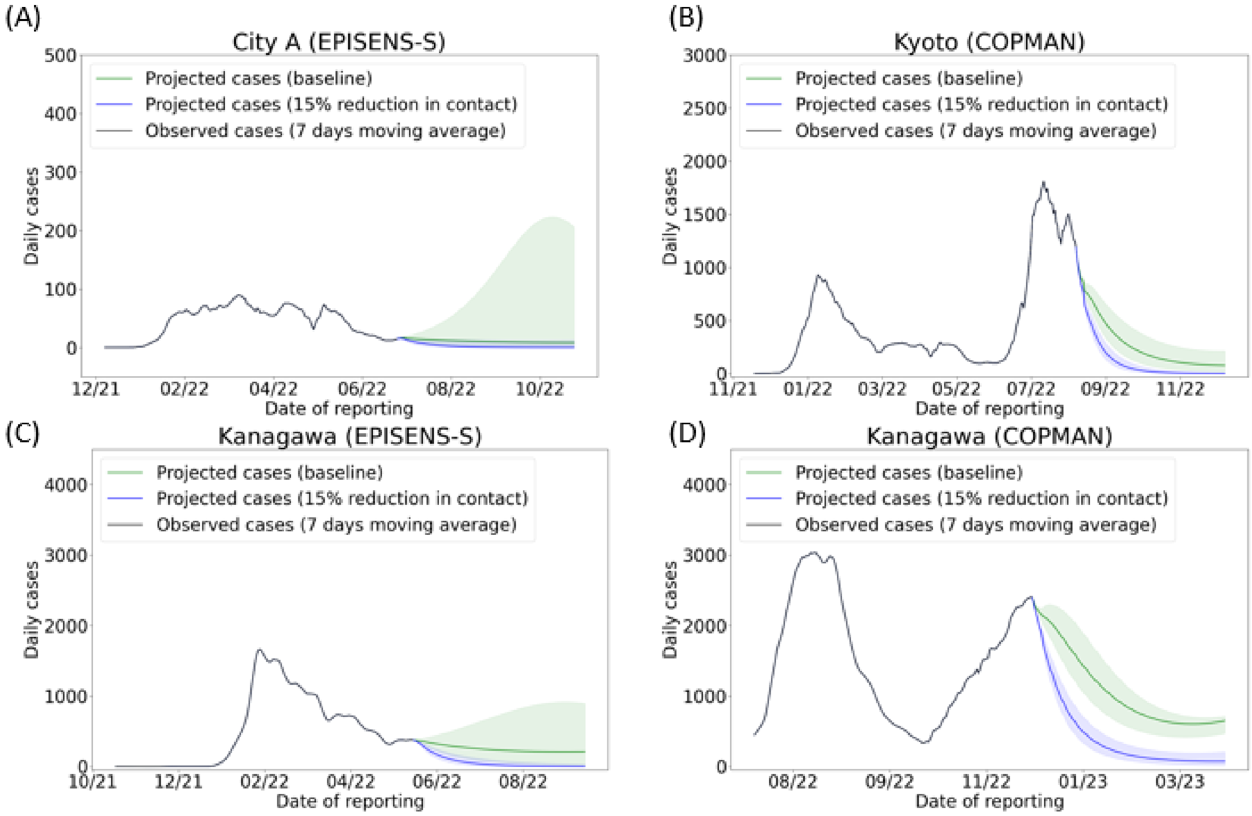

Figure S7. Additional analysis for a non-pharmaceutical intervention scenario. Relative contact rates are set as 1 for the baseline (green) and 0.85 (blue) and for scenarios with reduced contact rates. Each ribbon represents uncertainty ranges of 2 standard deviations (SD) computed by the estimated variance of the baseline transmission rate.

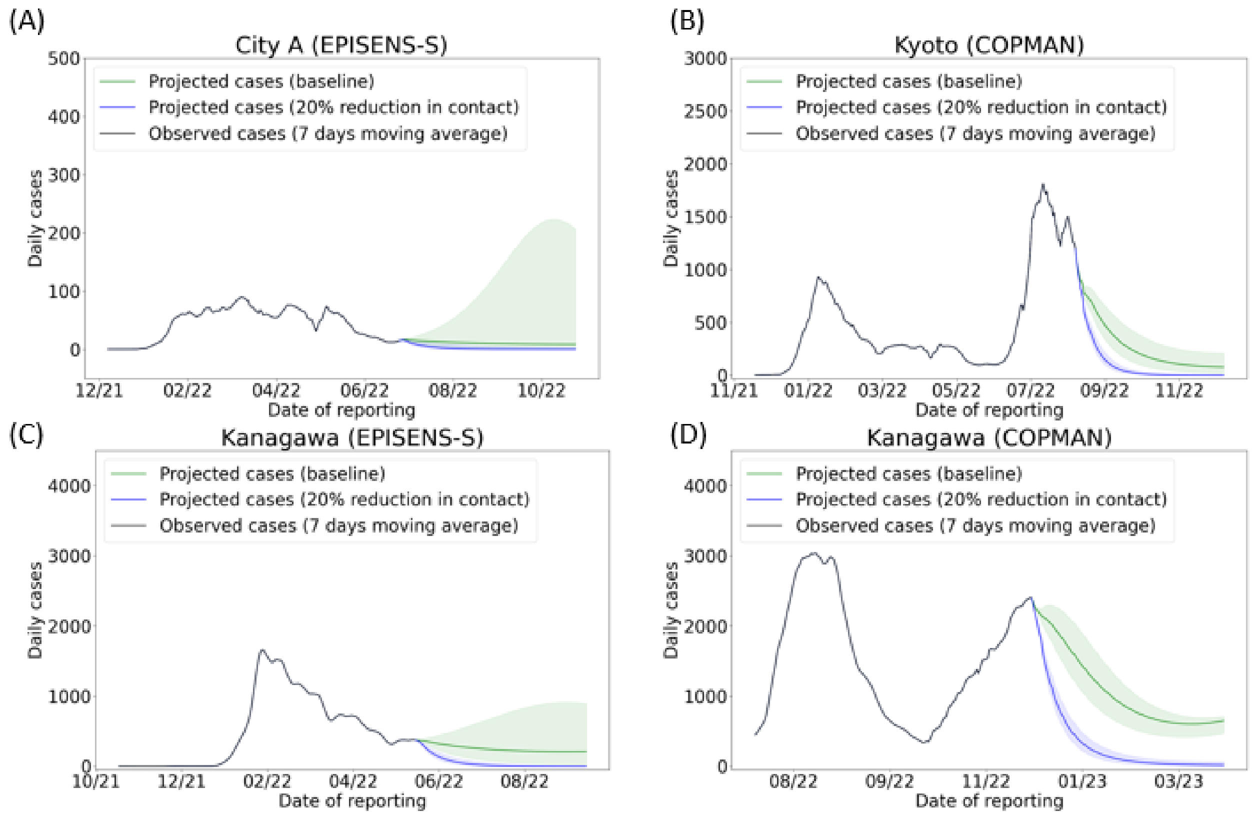

Figure S8. Model projected cases for a non-pharmaceutical intervention scenario. Relative contact rates are set as 1 for the baseline (green) and 0.8 (blue) and for scenarios with reduced contact rates. Each ribbon represents uncertainty ranges of 2 standard deviations (SD) computed by the estimated variance of the baseline transmission rate.

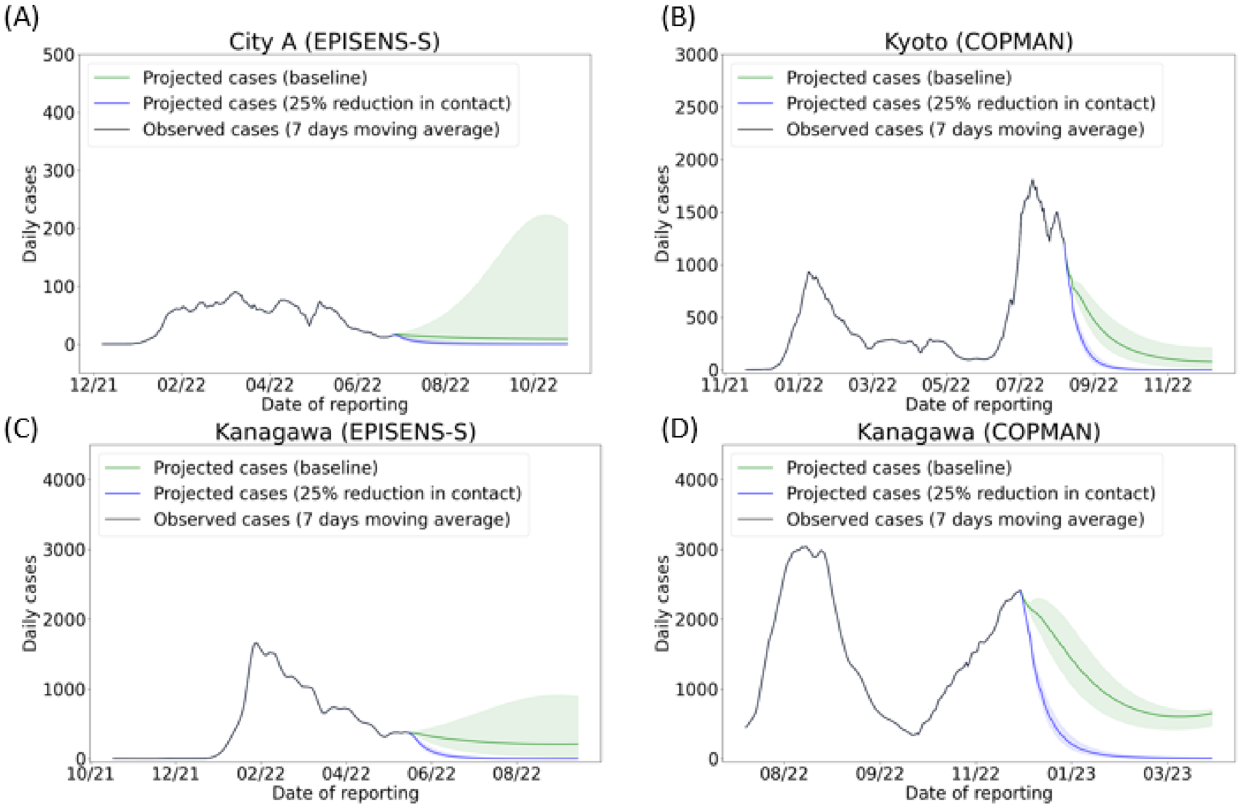

Figure S9. Model projected cases for a non-pharmaceutical intervention scenario. Relative contact rates are set as 1 for the baseline (green) and 0.75 (blue) and for scenarios with reduced contact rates. Each ribbon represents uncertainty ranges of 2 standard deviations (SD) computed by the estimated variance of the baseline transmission rate.

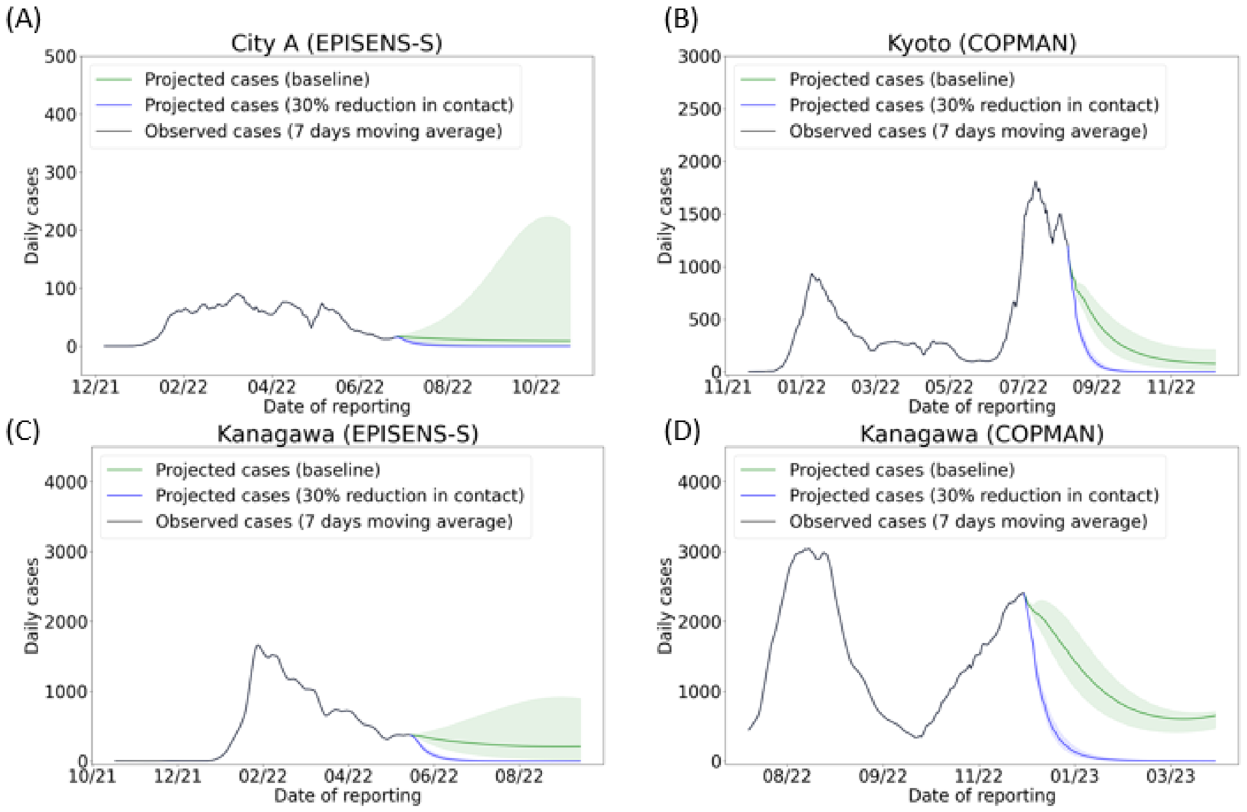

Figure S10. Model projected cases for a non-pharmaceutical intervention scenario. Relative contact rates are set as 1 for the baseline (green) and 0.7 (blue) and for scenarios with reduced contact rates. Each ribbon represents uncertainty ranges of 2 standard deviations (SD) computed by the estimated variance of the baseline transmission rate.

Table S1. Data source.

Municipalities	Wastewater treatment plant	Epidemiological data	Method	Period (Sampling frequency)	Wastewater data
Kyoto city	・Toba first WWTP<br>・Toba second WWTP	https://www.city.kyoto.lg.jp/hokenfukushi/page/0000268303.html	COPMAN	15 Dec 2021 ~ 28 Feb 2022 (3 times/week), 2 March 2022 ~ 31 Aug 2022 (2 times/week)	https://github.com/AdvanSentinel/AS-SEIRS
City A	・East WWTP	Not accessible to the general public	EPISENS-S	15 Dec 2021 ~ 30 Jun 2022 (2 times/week)	https://github.com/AdvanSentinel/AS-SEIRS
A part of Kanagawa (Sagami river basin)	・Right bank of the Sagami River WWTP<br>・Left bank of the Sagami River WWTP	http://covid-map.bmi-tokai.jp/choroplethmap_kanagawa/	EPISENS-S	1 Nov 2021 ~ 30 Mar 2022 (3 times /week), 19 Apr 2022 ~ 27 May 2022 (2 times/week)	https://www.pref.kanagawa.jp/docs/ga4/covid19/simulation.html, https://github.com/AdvanSentinel/AS-SEIRS
			COPMAN	8 Jul 2022 ~ 27 Dec 2022 (2 times/week)	https://github.com/AdvanSentinel/AS-SEIRS

Table S2. List of input parameters.

Parameter	Symbol	Kyoto city	City A	A part of Kanagawa (COPMAN)	A part of Kanagawa (EPISENS-S)
Sewered population size	N	778000	157113	1241200	
Latent period (days)	$1/\alpha$	1.5*			
Infectious period (days)	$1/\tau$	2.0†			
Immunity duration (days)	$1/\omega$	180††			
Special holidays		2021/1/1,2021/1/11,2021/2/11,2021/2/23,2021/3/20,2021/4/29,2021/5/3,2021/5/4,2021/5/5,2021/7/20,2021/8/8,2021/8/9,2021/9/20,2021/9/23,2021/11/3,2021/11/23,2022/1/10,2022/2/11,2022/2/23,2022/3/21,2022/4/29,2022/5/3,2022/5/4,2022/5/5,2022/7/18,2022/8/11,2022/9/19,2022/9/23,2022/10/10,2022/11/3,2022/11/23			

* this value refers to the start of infectious viral shedding for the Omicron variant [8]
† the mean infectious period is set as 2.0 days in order to fix the mean generation time of 3.5 days [9]
†† this value is defined as 180 days based on antibody titers measured among COVID-19 cases in Japan [10]

Table S3. List of estimated parameters.

**Parameter**	**Symbol**	**Kyoto city**	**City A**	**A part of Kanagawa (EPISENS-S)**	**A part of Kanagawa (COPMAN)**
Shedding duration (days)	$1/\gamma$	2.00	1.68	1.73	1.65
Scaling parameter	ν	2.5×10^{-11}	6.1×10^{-10}	4.2×10^{-11}	1.3×10^{-11}
Calibration period		15 Dec 2021 to 15 Jan 2022 (9 weeks)	15 Dec 2021 to 18 Jan 2022 (5 weeks)	1 Nov 2021 to 21 Mar 2022 (20 weeks)	8 July 2022 to 09 Sept 2022 (9 weeks)

Table S4. List of initial parameters.

**Parameter**	**Kyoto city**	**City A**	**A part of Kanagawa (EPISENS-S)**	**A part of Kanagawa (COPMAN)**
Seroprevalence*	0.95	0.95	0.95	0.89†
S (t=0)	739,100	149,258	1,179,140	1,110,566
E (t=0)	11	4	8	1,893
I (t=0)	14	5	10	2,524
R (t=0)	38,875	7,846	62,042	126,217

* this value is based on the estimated seroprevalence in a large scale serological survey of COVID-19 in Japan [11]
† this value refers to the estimated susceptible proportion in the model fit of Kanagawa EPISENS-S
